# Dimerized Translationally Controlled Tumor Protein-Binding Peptide 2 Attenuates Systemic Anaphylactic Reactions Through Direct Suppression of Mast Cell Degranulation

**DOI:** 10.3389/fphar.2021.764321

**Published:** 2021-10-19

**Authors:** Hyunsoo Cho, Jiyoung Park, Hyo Kyeong Kim, Eun Sook Hwang, Kyunglim Lee

**Affiliations:** ^1^ Graduate School of Pharmaceutical Sciences, College of Pharmacy, Ewha Womans University, Seoul, South Korea; ^2^ Fluorescence Core Imaging Center, Department of Life Science, Ewha Womans University, Seoul, South Korea

**Keywords:** anaphylaxis, BMMCs, calcium influx, compound 48/80, DTBP2, dTCTP

## Abstract

Dimerized translationally controlled tumor protein (dTCTP) amplifies allergic responses through activation of several types of immune cells and release of inflammatory mediators. In particular, dTCTP plays an important role in histamine release by triggering mast cells and has been proposed as a target in the treatment of allergic diseases. dTCTP-binding peptide 2 (dTBP2) is known to attenuate severe allergic rhinitis and asthma through inhibition of dTCTP activity on airway epithelial cells and T cells; however, it is unclear whether dTBP2 affects mast cell function and mast cell disease. In this study, we explored the effects of dTBP2 on mast cell degranulation and allergen-induced anaphylactic reactions. We found that bacterial product lipopolysaccharide increased the expression of dTCTP in mast cells and rapidly released dTCTP by the mast cell stimulator compound 48/80. Interestingly, the released dTCTP further promoted mast cell degranulation in an autocrine activation manner and increased calcium mobilization in mast cells, which is essential for degranulation. Furthermore, dTBP2 directly and dose-dependently inhibited *in vitro* mast cell degranulation enhanced by compound 48/80, suggesting a direct and potent anti-anaphylactic activity of dTBP2. dTBP2 also significantly suppressed the dTCTP-induced degranulation and histamine release through inhibition of the p38 MAPK signaling pathway and suppression of lysosomal expansion and calcium mobilization in mast cells. More importantly, *in vivo* administration of dTBP2 decreased mortality and significantly attenuated histamine release and inflammatory cytokine production in compound 48/80-induced systemic anaphylactic reactions. These results suggest that dTBP2 is beneficial for the control of anaphylaxis with increased dTCTP.

## Highlights

• The dTCTP inhibitor, dTBP attenuates the anaphylactic reactions
• dTBP2 suppresses mast cell degranulation *in vitro*
• dTCTP is rapidly secreted by activated mast cells causing mast cell degranulation through an autocrine activation pathway
• dTBP2 inhibits lysosomal expansion and calcium mobilization increased by dTCTP.

## Introduction

Anaphylaxis is a severe and potentially life-threatening allergic response and occurs rapidly after exposure to provoking agents (Bochner and Lichtenstein, 1991). Anaphylaxis, also known as anaphylactic shock, is caused by an overreaction of most immune cells and is characterized by airway constriction, circulatory collapse, and mucosal inflammation, leading to difficulty breathing and death (Reber et al., 2017). Activation of potential effector cells, mainly mast cells, immediately releases mediators such as histamine and various proteases and pro-inflammatory cytokines and subsequently promotes recruitment of type 2 T helper cells, activation of dendritic cells and airway epithelial cells, thereby exacerbating allergic disorders (Kraft and Kinet, 2007; Galli and Tsai, 2012). Therapeutic approaches to anaphylaxis such as adrenaline as first-line treatment, glucagon as second-line treatment, and anti-histamines as third-line treatment have long been attempted and developed; however, there are still unmet therapeutic needs for anaphylaxis (Tanno et al., 2019). A better understanding of the mechanisms and causes of anaphylaxis will help develop therapeutics based on precision medicine. Antigen-specific IgE antibodies and FcεR1-bearing effector cells, mainly mast cells, play a dominant role in anaphylaxis through the production of mediators (Ishizaka et al., 1972). Anaphylaxis is caused by IgE-mediated mast cell degranulation and IgE-independent mechanisms such as IgG-dependent anaphylaxis, complement-mediated anaphylaxis, and drug-induced anaphylaxis (Finkelman et al., 2016). Since compound 48/80 (C48/80) specifically induces mast cell activation and degranulation, it is widely used as a potent histamine liberator in animal and tissue models of systemic anaphylaxis (Paton, 1951; Wang et al., 2020).

Translationally controlled tumor protein (TCTP) is also known to be an IgE-dependent histamine-releasing factor (HRF) because of its cytokine-like activity (MacDonald et al., 1995). TCTP regulates many cellular events in either the monomeric (mTCTP) or dimerized form (dTCTP) in intracellular and extracellular compartments (Kaplan et al., 1991; Kim et al., 2009; Macdonald, 2012; Kawakami et al., 2019). While mTCTP plays a role in the regulation of cell proliferation, survival, malignant transformation, and epithelial-mesenchymal transition within the cells, extracellular dTCTP triggers histamine release and increases the secretion of pro-inflammatory cytokines by basophils, mast cells, and epithelial cells (Bae et al., 2015; Lee and Lee, 2018; Ulambayar et al., 2019; Bommer and Telerman, 2020; Lee et al., 2020). Extracellular dTCTP or HRF stimulates histamine release and secretion of IL-4 and IL-13 from human basophils, induces IL-8 from bronchial epithelial cells, and promotes IL-4, IL-5, and IL-13 secretion from Th2 cells (Schroeder et al., 1996; Kashiwakura et al., 2012; Kawakami et al., 2019; Kim et al., 2021). HRF also binds to a specific subset of membrane IgE molecules and enhances production of IL-1β and IL-6 and B cell proliferation (Kang et al., 2001). Elevated dTCTP or HRF production is associated with the increased inflammatory cytokines in the body fluids of patients with allergic diseases such as atopic dermatitis, food allergies, and asthma (Sampson et al., 1989; Kim et al., 2009; Valenta et al., 2009; Kashiwakura et al., 2012; Lommatzsch and Virchow, 2014; Ando et al., 2017). Therefore, dTCTP has been proposed as a therapeutic target for treating allergic diseases. Indeed, a dTCTP inhibitor, dTCTP-binding peptide 2 (dTBP2), has been reported to block dTCTP activity and attenuate allergic rhinitis, atopic dermatitis, and rheumatoid arthritis (Kim et al., 2011; Jin et al., 2017; Kim et al., 2021).

In this study, we explored the effects of dTBP2 on *in vivo* anaphylactic reactions and *in vitro* mast cell degranulation, and further analyzed the molecular mechanisms underlying the inhibition of dTBP2 in mast cell degranulation.

## Materials and Methods

### Reagents

Compound 48/80 (C48/80) was purchased from Sigma-Aldrich (St. Louis, MO, United States). The Dulbecco’s modified Eagle’s medium (DMEM) and fetal bovine serum (FBS) were purchased from Thermo Fisher Scientific (Waltham, MA, United States). Recombinant TCTP proteins r-dTCTP and r-mTCTP were isolated from the BL21 (DE3) PLysS strain that was transformed with PRSET A vector of His-tagged dTCTP and mTCTP. r-dTCTP and r-mTCTP were used for cell treatment after purification using Ni-NTA agarose (QIAGEN, Hilden, Germany) and anion Exchange Chromatography with HiTrap Q Column (GE Healthcare Bio-Sciences Corp, Piscataway, NJ, United States), followed by endotoxin removal. dTBP2 peptide was synthesized at the peptide synthesizer facility PepTron Inc. (Daejeon, Korea) and the purity of the synthetic peptide was confirmed to be 98%.

### Isolation and Cultivation of Bone-Marrow Derived Mast Cells)

Bone marrow cell suspensions were isolated by flushing femurs and tibias from C57BL/6J mice (male, 8 weeks old), and cultured in RPMI-1640 supplemented with 10% FBS, 1% penicillin-streptomycin, 50 μM 2-mercaptoethanol, and recombinant murine IL-3 (rmIL-3, 10 ng/ml, BioLegend, San Diego, CA, United States) for 4 weeks (Liao et al., 2020). The purity of BMMCs assessed by staining and flow cytometry analysis with anti-FcεRI (Invitrogen, Carlsbad, CA, United States) and anti-c-kit antibody (eBioscience, San Diego, CA, United States) confirmed that they are 95% pure. The BMMCs were cultivated between days 29 and 49 post-isolation and were used for all functional assays.

### β-Hexosaminidase Assay

Untreated BMMCs or those pre-treated with vehicle, r-dTCTP, and r-mTCTP for 24 h were centrifuges and the cell pellets were resuspended in extracellular buffer (10 mM HEPES, 137 mM NaCl, 2.7 mM KCl, 0.4 mM Na_2_HPO4.7H2O, 1.4 mM CaCl_2_, 1 mM MgCl_2_, 5.6 mM glucose, and 0.04% BSA), and treated with 50 μg/ml C48/80 for either 5 min or indicated time. Untreated sample were prepared to use as controls. The cell supernatant was harvested and subjected to β-hexosaminidase release assay using 4-nitrophenyl 2-acetamido-2-deoxy-β-D-glucopyranoside in 0.1 M citrate buffer (pH 4.2) (PNAG, 3.4 mg/ml, TCI Chemicals, Japan). Reaction was stopped with 0.4 M glycine buffer (pH 10.8), and optical density was measured at 405 nm.

### Immunofluorescence and LysoTracker Staining

BMMCs were incubated with LPS (1 µg/ml) for 24 h and treated with C48/80 (50 µg/ml) for 5 min before harvest. Cells were fixed with 4% paraformaldehyde for 10 min at RT, permeabilized in 0.1% Triton X-100/PBS for 10 min, and incubated with antibody against lysosomal membrane-associated protein 1 (LAMP1, Santa Cruz Biotech Inc., Santa Cruz, CA, United States) and TCTP (Abcam, Cambridge, MA, United States), followed by staining with Alexa Fluor 488- or Alexa Fluor 555-coupled secondary antibodies (Invitrogen). Nuclei were stained with DAPI (1 µg/ml). Cells were observed under a confocal laser scanning microscope (ZEISS LSM 880 with Airyscan, Oberkochen, Germany). For LysoTracker staining, cells were pre-treated with dTCTP (10 µg/ml) and/or dTBP2 (100 µg/ml) and incubated with LysoTracker™ (75 nM, Thermo Fischer Scientific) for 1 h at 37°C in dark. Cells were fixed with 4% PFA and observed under a confocal microscope equipped at Drug Development Research Core Center.

### ELISA

BMMCs (1 × 10^6^ cells/ml) were incubated with the vehicle, r-dTCTP, or r-mTCTP in the presence or absence of dTBP2 for 24 h. The culture supernatants were collected and analyzed with commercial ELISA kits according to the manufacturer’s instructions. Histamine level in serum was measured with histamine EIA kit (Oxford biomedical research, Rochester Hills, MI, United States) and serum TNF-α and IL-6 level was measured with murine TNF-α and IL-6 ELISA kit (BioLegend).

### Giemsa Staining

BMMCs were incubated with vehicle, r-dTCTP, r-mTCTP in the presence or absence of dTBP2 (100 μg/ml) for 24 h, and centrifuged (Cytospin 4, Thermo Fisher Scientific) and subjected to Giemsa staining (Diff-Quik staining kit, Sysmex Co., Kobe, Japan). BMMCs were also incubated with either vehicle or dTBP2 (100 μg/ml) in the presence of C48/80 (20 μg/ml) and then applied on slide glass with Cytospin 4, and stained with Diff Quik Solution.

### Immunoblot Analysis

BMMCs were harvested and extracted with RIPA lysis buffer. Protein extracts and culture supernatants were resolved by SDS-polyacrylamide gel electrophoresis, followed by electrotransfer. Protein blots were incubated with antibody against TCTP (Abcam), Phospho-NF-κB p65, NF-kB p65, Phospho-p38, p38, Phospho-ERK, ERK (Cell signaling technology, Danvers, MA, United States), and β-actin (bs-0061R, 1:5000, Bioss, Woburn, MA, United States), and protein signal was visualized with Image Analyzer (ChemiDoc MP, Bio-Rad, Hercules, CA, United States).

### Calcium Influx Assay

BMMCs were plated into black wall, transparent bottom 96-well plates (BD, Franklin Lakes NJ, United States) and pre-treated with vehicle, LPS, r-dTCTP (10 µg/ml) or r-mTCTP (10 µg/ml) in the presence or absence of dTBP2 (100 µg/ml) for 24 h. The cells were then assayed for calcium with Fluo-4 Direct Calcium Assay Kit (Thermo Fisher Scientific), followed by measurement using Tecan infinite F200 fluorescence microplate reader (Tecan, Männedorf, Switzerland). After 30 min incubation, cells were additionally treated with C48/80 (20 µg/ml) and measured with fluorescence microplate reader for 5 min. The basal fluorescence intensity was subtracted to calculate the relative fluorescence intensity over time.

### C48/80-Induced Systemic Anaphylaxis Animal Model

C57BL/6J mice (male, 8 weeks old) were obtained from OrientBio Inc. (Seongnam, Korea), and were housed at a temperature of 22 ± 1°C and relative humidity of 55 ± 10%. Mice (males, 8 weeks old) were injected intraperitoneally with PBS or C48/80 (8 mg/kg) freshly dissolved in PBS. Low (0.3 mg/kg) and high doses (1 mg/kg) dTBP2 or dexamethasone (DXM, 4 mg/kg) were injected into the animals 15 min before C48/80. Mortality rate was monitored for 1 h after induction of systemic anaphylactic reactions by C48/80. Blood samples were collected through heart puncture to measure the levels of histamine, TNF-α, and IL-6. All animal experiments were conducted in accordance with the international guidelines approved by Ewha Womans University’s Institutional Animal Care and Use Committee (IACUC 18-040).

### Statistical Analysis

All data are expressed as the mean ± SEM. Results were analyzed by one-way ANOVA or two-tailed Student’s t-test. *p* values less than 0.05 were considered to indicate a statistically significant difference.

## Results

### LPS-Induced dTCTP is Rapidly Released by Mast Cells Upon C48/80 Stimulation and Enhances Mast Cell Degranulation *via* an Autocrine Activation Pathway

**FIGURE 1:**
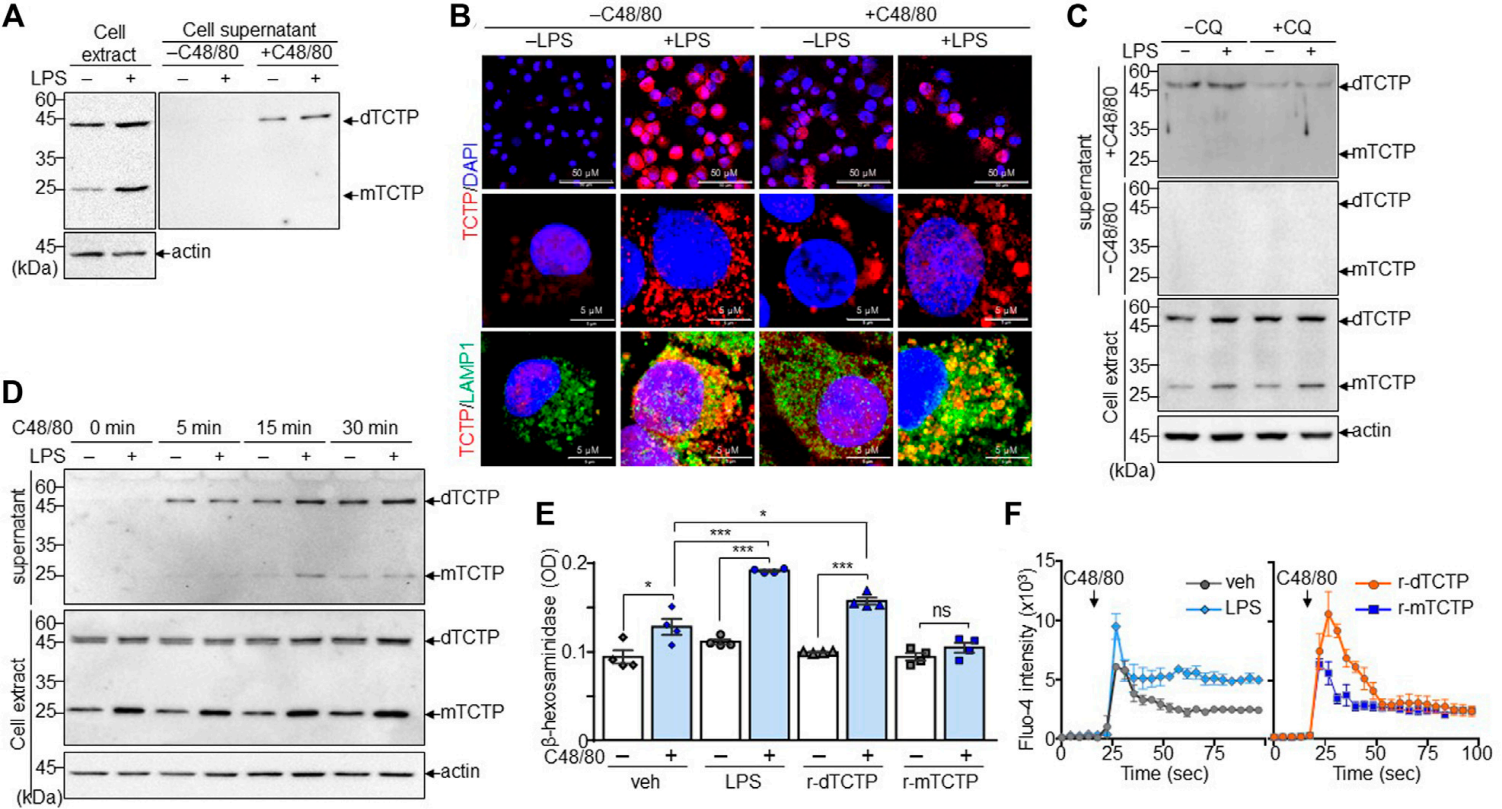
Mast cell degranulation by the dTCTP-mediated autocrine activation. BMMCs were stimulated with LPS (1 µg/ml) for 24 h and subsequently incubated with or without treatment of C48/80 (50 µg/ml) for 5 min. Cells and culture supernatants were collected for the further analysis. **(A)** Immunoblot analysis of intracellular and extracellular TCTP. **(B)** Immunofluorescence staining of endogenous TCTP and LAMP-1. DAPI was used for nuclear staining (Scale bar: 5 µm or 50 µm). **(C)** Cells were treated with LPS or 24 h and incubated with or without CQ (20 μM) for 4 h before harvest. Cell extracts and supernatant were analyzed by immunoblot analysis. **(D)** BMMCs treated with either vehicle or LPS (1 µg/ml) were subsequently treated with C48/80 (50 µg/ml) for the indicated time points. Cell extracts and supernatants were subjected to immunoblot analysis. **(E)** BMMCs were treated with vehicle, LPS, r-dTCTP, or r-mTCTP and additionally incubated with C48/80 (50 µg/ml) for 5 min, followed by the β-hexosaminidase assay. *n* = 4. ns, not significant; *, *p* < 0.05; and ***, *p* < 0.0005 by Student’s *t*-test. **(F)** Calcium influx assay was conducted with Fluo-4 dye in BMMCs. *n* = 5. Representative image from five independent experiments is presented in **(A–D)**. Data in **(E)** and **(F)** are given as the mean ± SEM.

Since dTBP2 has been identified as a specific inhibitor for dTCTP, which is primarily produced by mononuclear cells and released under allergic conditions, we first asked whether mast cells produce dTCTP and dTCTP modulates mast cell function. Both dTCTP and mTCTP were strongly expressed in mast cells, which were further increased by LPS stimulation (Figure 1A). Interestingly, dTCTP stably expressed as a dimer even under reducing conditions, and intracellular TCTP was rarely detected in the extracellular compartment. But dTCTP secretion was increased by treatment with C48/80 (Figure 1A). Immunofluorescence staining confirmed that intracellular TCTP expression was markedly increased by LPS, mostly co-localized with LAMP1 in the cytoplasm, and rapidly released as secretory lysosomes by C48/80 treatment (Figure 1B). In addition, the release of dTCTP into the extracellular compartment was inhibited by treatment with the lysosomal inhibitor, chloroquine (CQ) (Figure 1C). Furthermore, time-course analysis of dTCTP secretion by C48/80 treatment demonstrated a rapid release of dTCTP within 5 min after C48/80 treatment and persistent release of dTCTP during mast cell degranulation induced by C48/80 (Figure 1D). We thus purified endotoxin-free recombinant proteins r-dTCTP and r-mTCTP and investigated whether extracellular recombinant TCTP proteins affect mast cell degranulation. To prevent overwhelming effect of C48/80, BMMCs were incubated with LPS, r-dTCTP, or r-mTCTP for 24 h and briefly treated with C48/80 to induce mast cell degranulation. C48/80 treatment slightly increased β-hexosaminidase release through mast cell degranulation, and LPS dramatically increased mast cell degranulation. In addition, while r-mTCTP treatment had no effect on β-hexosaminidase release by BMMCs, r-dTCTP significantly increased mast cell degranulation (Figure 1E), indicating that dTCTP is a major mediator of LPS-induced mast cell degranulation. Furthermore, intracellular calcium mobilization triggered by C48/80 was further increased in LPS-stimulated BMMCs than in vehicle-treated cells. Likewise, r-dTCTP treatment enhanced intracellular calcium mobilization in BMMCs, whereas r-mTCTP showed similar levels compared to vehicle-treated BMMCs (Figure 1F). These results suggest that dTCTP is rapidly released by activated mast cells and further potentiates mast cell degranulation *via* an autocrine activation loop.

### dTBP2 Directly and Potently Suppresses Mast Cell Degranulation *In Vitro*

**FIGURE 2:**
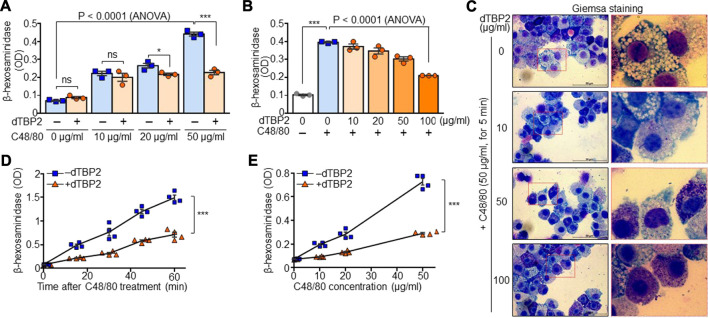
Potent inhibition of mast cell degranulation by dTBP2. **(A)** BMMCs were incubated with dTBP2 (100 µg/ml) for 24 h and additionally treated with C48/80 for 30 min. Cells were resuspended in assay buffer and the supernatants were used for measuring β-hexosaminidase activity. *n* = 3. ns, not significant; *, *p* < 0.05; **, *p* < 0.005; and ***, *p* < 0.0005 by Student’s *t*-test. *p* < 0.0001 by ANOVA. **(B, C)** BMMCs were treated with the indicated amounts of dTBP2 for 24 h. Cells were resuspended in assay buffer and incubated with C48/80 (50 µg/ml) for 30 min and subjected to the β-hexosaminidase assay **(B)** and Giemsa staining **(C)**. *n* = 3. ***, *p* < 0.0005 by Student’s *t*-test. *p* < 0.0001 by one-way ANOVA. Representative image of five independent experiments is presented in **(C)**. **(D)** BMMCs were re-suspended in assay buffer and incubated with C48/80 in the presence or absence of dTBP2 (100 μg/ml) for the indicated time points at room temperature. β-hexosaminidase activity was determined from the supernatants. *n* = 4. ***, *p* < 0.0005 by Student’s *t*-test. **(E)** BMMCs resuspended in assay buffer were incubated with different concentrations of C48/80 in the presence or absence of dTBP2 (100 μg/ml) for 30 min at room temperature, followed by the β-hexosaminidase assay. *n* = 4. ***, *p* < 0.0005 by Student’s *t*-test. Data in **(A, B, D, E)** are given as the mean ± SEM.

We next investigated whether dTBP2 directly attenuates potentiated degranulation of BMMCs by prolonged triggering with C48/80 *in vitro*. β-Hexosaminidase activity, the indication of mast cell degranulation, increased in BMMCs in a C48/80 dose-dependent manner, and pretreatment of BMMCs with dTBP2 significantly decreased mast cell degranulation potentiated by C48/80 (Figure 2A). In addition, dTBP2 dose-dependently decreased β-hexosaminidase release in BMMCs induced by C48/80 (Figure 2B). Giemsa staining also demonstrated that mast cell degranulation was increased by C48/80 but was instantly inhibited by the addition and increase of dTBP2 during mast cell degranulation (Figure 2C). Moreover, *in vitro* degranulation of BMMCs was time-dependently increased with prolonged incubation with C48/80, and the transient incubation with dTBP2 during mast cell degranulation without pretreatment effectively inhibited mast cell degranulation (Figure 2D). The potent inhibitory activity of dTBP2 on *in vitro* mast cell degranulation was also confirmed at various C48/80 concentrations (Figure 2E). These results indicate that dTBP2 directly and effectively inhibits antigen-induced degranulation of mast cells.

### dTBP2 Blocks dTCTP-Mediated Autocrine Activation Loop in Mast Cells and Inhibits p38 Signaling Pathway and Lysosomal Expansion

**FIGURE 3:**
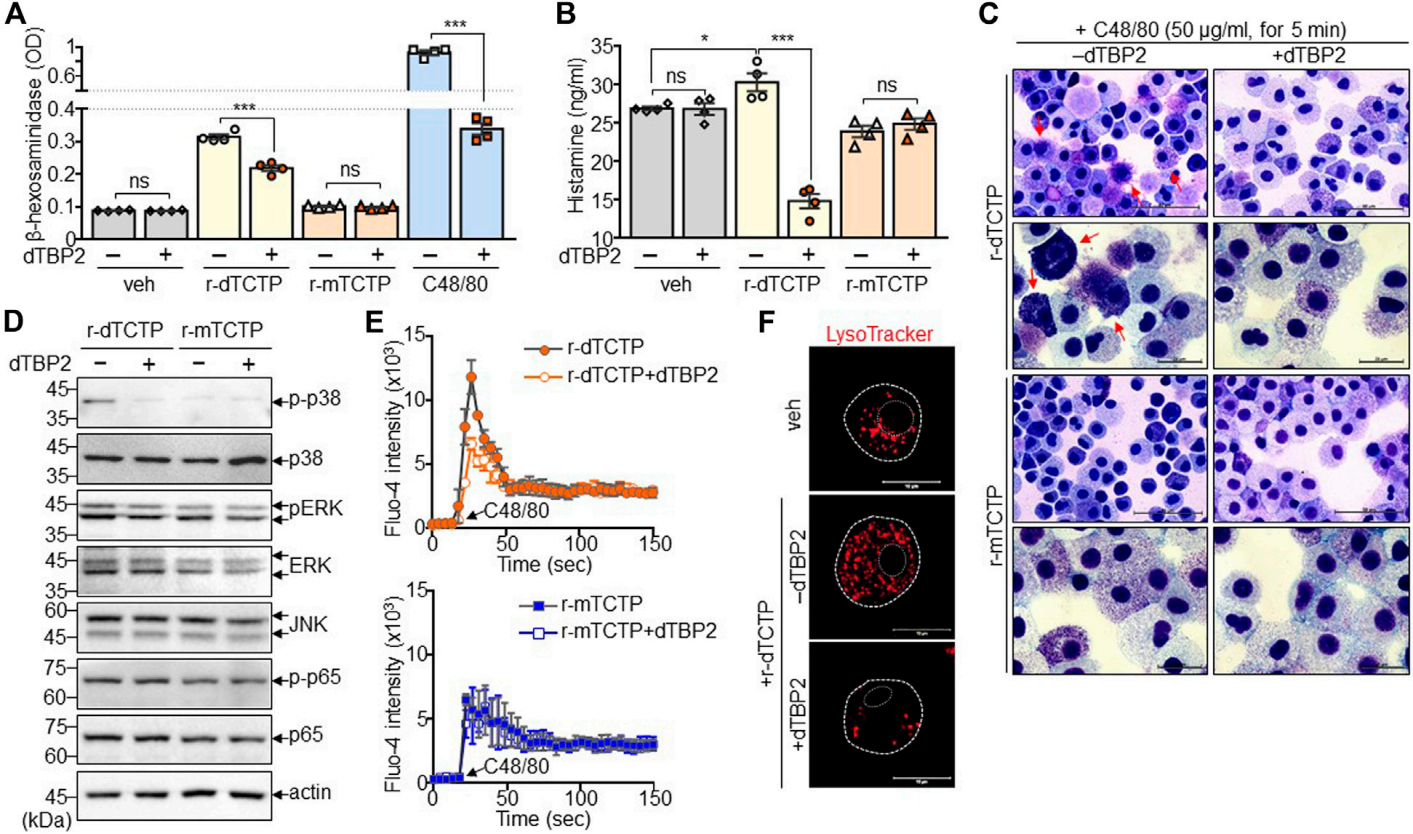
Suppression of dTCTP-induced p38 MAPK activation and lysosomal expansion by dTBP2. BMMCs were incubated with either r-dTCTP (10 μg/ml) or r-mTCTP (10 μg/ml) in the presence or absence of dTBP2 (10 μg/ml) for 24 h and harvested for further analysis. **(A)** Cells were harvested and subjected to the β-hexosaminidase assay. As a control for the β-hexosaminidase assay, cells were incubated with C48/80 (50 μg/ml) for 30 min prior to the assay. *n* = 4. ns, not significant; and ***, *p* < 0.0005 by Student’s *t*-test. **(B)** Cell supernatants were subjected to histamine ELISA. *n* = 4. ns, not significant; *, *p* < 0.05; and ***, *p* < 0.0005 by Student’s *t*-test. **(C)** Cells were subjected to Giemsa staining and microscopic observation. **(D)** Cell extracts were subjected to immunoblot analysis. **(E)** Calcium influx using Fluo-4 dye was conducted in BMMCs. *n* = 5. **(F)** Cells were stained with LysoTracker and observed under a fluorescence microscope. Data in **(A, B, E)** are given as the mean ± SEM. Representative image of five independent experiments is presented in **(C, D, F)**.

**FIGURE 4:**
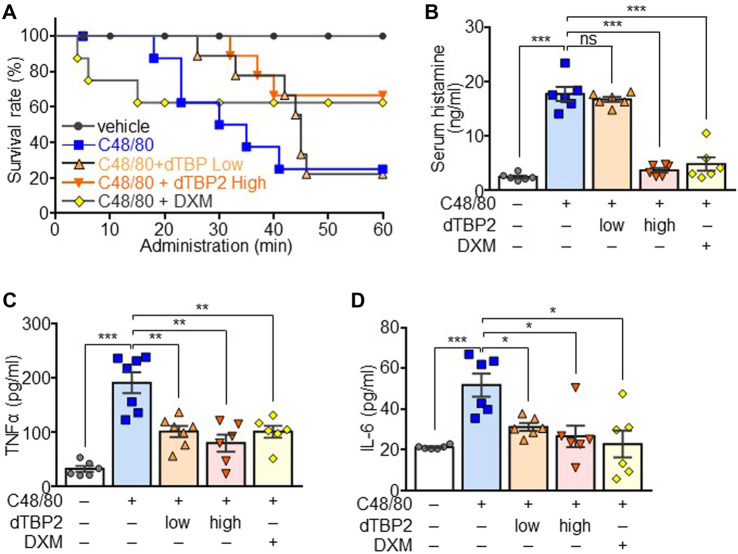
Attenuation of the C48/80-induced anaphylactic reactions by dTBP2. Wild-type C57BL6 mice (males, 8 weeks old) were divided into 5 groups (*n* = 8 per group). Group I was injected with only PBS and used as a control group. Groups II, III, and IV received vehicle, 0.3 mg/kg **(low)** dTBP2, and 5 mg/kg **(high)** dTBP2, respectively, *via* the intravenous route. Group V received intraperitoneal DXM (4 mg/kg) injection. Groups II–V were subsequently injected with C48/80 after 15 min and monitored for 1 h. **(A)** Survival rate was determined by calculation of the percent survival at each time point using Graphpad. *n* = 8. **(B–D)** Serum samples were collected from each group and subjected to measure histamine release **(B)**, TNF-α **(C)**, and IL-6 **(D)** by ELISA. Data are expressed as the mean ± SEM. ns, not significant; *, *p* < 0.05; **, *p* < 0.005; and ***, *p* < 0.0005 by Student’s *t*-test.

We next attempted to confirm the dTCTP-dependent inhibitory activity of dTBP2 and investigated its underlying molecular mechanisms. As previously confirmed, dTBP2 substantially inhibited mast cell degranulation potentiated by prolonged treatment or high concentration of C48/80. In addition, the release of β-hexosaminidase was increased by r-dTCTP and significantly decreased by dTBP2 addition in BMMCs, whereas r-mTCTP did not induce mast cell degranulation (Figure 3A). Moreover, histamine release by BMMCs was also significantly increased by r-dTCTP but not r-mTCTP, and dTCTP-induced histamine release was markedly decreased by dTBP2 treatment (Figure 3B). Extracellular r-dTCTP, not r-mTCTP, treatment increased the formation of dense purple granules within BMMCs and instantly induced mast cell degranulation. The dTCTP-induced mast cell granule formation and degranulation was impaired in the presence of dTBP2, which was evidenced by Giemsa staining (Figure 3C). Further analysis confirmed that phosphorylation of p38 MAPK was increased by r-dTCTP but not affected by r-mTCTP. dTBP2 treatment inhibited the r-dTCTP-induced p38 MAPK signaling pathway essential for mast cell degranulation, but did not affect the NF-kB signaling pathway (Figure 3D). In addition, intracellular calcium mobilization increased by r-dTCTP was decreased by treatment with dTBP2, but there was no change in r-mTCTP-treated mast cells (Figure 4E). Interestingly, expansion and acidification of lysosomal compartments essential for anaphylactic degranulation (Pejler et al., 2017) were prominently found in r-dTCTP-treated BMMCs but were consequently decreased by addition of dTBP2 (Figure 3F). Our results indicate that the anti-anaphylactic activity of dTBP2 is due to the specific inhibition of p38 MAPK activation and calcium mobilization in mast cells activated by autocrine dTCTP signaling.

### C48/80-Induced Systemic Anaphylactic Reactions are Suppressed by dTBP2 in Mice

To evaluate the *in vivo* anti-anaphylactic effect of dTBP2, an animal model for systemic anaphylaxis was established by intraperitoneal injection of C48/80 into mice and additionally administered low-dose and high-dose dTBP2 through intravenous injection. Most mice died within 1 h after C48/80 injection, and mortality reached 80%. Low-dose dTBP2 delayed morality but had no effect on C48/80-induced mortality, whereas high-dose dTBP2 significantly delayed and improved mortality (Figure 4A). DXM injection reduced C48/80-induced mortality, which was similar to the effect of high-dose dTBP2. Histamine release induced by C48/80 injection was decreased in the high-dose dTBP2 injection group as in the DXM-treated group, whereas there was no significant change in histamine release in the low-dose dTBP2 injection group (Figure 4B). Consistently, serum levels of pro-inflammatory cytokines TNF-α and IL-6 were elevated in C48/80-induced anaphylaxis mouse models but were significantly reduced by treatment with DXM. Both low-dose and high-dose dTBP2 also markedly decreased the production of pro-inflammatory cytokines (Figures 4C,D). These results indicate that dTBP2 is effective in inhibiting the C48/80-induced anaphylactic reactions *in vivo*.

## Discussion

We demonstrated that dTBP2, known as a dTCTP inhibitor, potently and efficiently suppressed the C48/80-induced anaphylactic reactions *in vivo*. We further demonstrated dTCTP was rapidly released from activated mast cells and promoted degranulation of mast cells with increased calcium mobilization and lysosomal expansion, and that this autocrine activation loop induced by dTCTP was blocked by dTBP2 in BMMCs. Therefore, dTBP2 is proposed as a potent anti-anaphylactic agent against pro-anaphylactic function of dTCTP.

Anaphylaxis is a severe allergic reaction that is rapid in onset and may cause multiple symptoms, including an itchy rash, throat or tongue swelling, short breathing, vomiting, low blood pressure, and even death (Finkelman et al., 2016; Jimenez-Rodriguez et al., 2018). It is commonly caused by insect bites and stings, food, and medications with multi-system involvement (Jonsson et al., 2019). Serious anaphylactic reactions have recently been reported with influenza vaccination (McNeil and DeStefano, 2018) and SARS-CoV-2 virus infection (Conti et al., 2020). Although there have been some reports that the first dose of the COVID-19 vaccine causes allergic reactions, including anaphylaxis, severe allergic reactions have been identified primarily in patients with a history of allergy or anaphylaxis and have occurred very rarely (Bellomo et al., 2021; Shimabukuro and Nair, 2021; Turner et al., 2021). Anaphylactic pathogenesis is driven by the release of inflammatory mediators and cytokines from certain types of immune cells triggered by allergens, causing symptoms in various tissues and organs (Khan and Kemp, 2011; Reber et al., 2017). In general immunologic mechanisms, allergens induce the activation of mast cells and basophils through cross-linking of IgE and its receptors and lead to the release of inflammatory mediators such as histamine. These mediators subsequently affect many body systems, including bronchial smooth muscle contraction, vasodilation, and heart muscle depression (Finkelman et al., 2016; Reber et al., 2017). Moreover, IgG antibodies or complement have been reported to mediate immunologic pathogenesis of anaphylaxis in mouse models, but are not yet clear in humans (Jonsson et al., 2019). Anaphylaxis also occurs in non-immunologic mechanism through the direct stimulation of mast cell degranulation by some drugs such as non-steroidal anti-inflammatory drugs, antibiotics, and opioids (Montanez et al., 2017). C48/80 is a mixed polymer of ρ-methoxy-N-methyl phenylethylamine cross-linked by formaldehyde and explicitly induces mast cell activation and histamine release in a non-immunologic mechanism. Therefore, C48/80 has been widely used for *in vitro* mast cell degranulation and *in vivo* animal models of anaphylaxis (Paton, 1951; Chatterjea et al., 2012). Here, we evaluated the effects of dTBP2 on the C48/80-induced systemic anaphylactic reactions to verify its specific function in mast cells.

The understanding of multifaceted immune cell function and the pivotal role of MCs in anaphylactic reactions has led to significant advances in the development of therapeutics, including anti-histamines, anti-IgE antibody, and leukotriene antagonists. Despite many clinical trials and efforts for the treatment of anaphylaxis, there is still need to develop effective therapeutics for anaphylaxis with multi-system interventions. It is possible to develop anti-anaphylaxis drugs that act in the regulation of mast cell degranulation process, production of mediators in granules, and receptor signaling in mast cell activation. In this study, we demonstrated that dTCTP was rapidly released from BMMCs upon C48/80 stimulation and further promotes mast cell degranulation in an autocrine manner. Thus, a dTCTP-specific inhibitor, dTBP2 potently attenuated mast cell degranulation *in vitro* and *in vivo*, which may have been mediated through inhibition of the autocrine activation pathway. dTBP2 is proposed as a potent therapeutic candidate for anaphylactic shock acting on the facilitated mast cell degranulation process. Furthermore, therapeutics strategies to disrupt lysosomal functions may be beneficial for blocking mast cell-mediated anaphylactic reactions through production of non-functional mediators (Moon et al., 2014; Krystel-Whittemore et al., 2015). Indeed, hydroxyl-CQ was reported to interfere with lysosomal function and accumulates non-functional tryptase in the mast cell granules, leading to decrease in the production of pro-inflammatory mediators (Espinosa et al., 2018). We also demonstrated that dTCTP was released *via* a secretory lysosome and its release was inhibited by the presence of CQ. Extracellular dTCTP facilitated lysosomal expansion of mast cells, contributing to mediator liberation, suggesting an interdependence between dTCTP production and lysosomal functions, distribution and dynamics in mast cells. Lysosomal expansion is generally increased by CQ due to the blockade of the lysosomal-autophagy pathway (Mauthe et al., 2018), but histamine release and mast cell degranulation are inhibited by CQ (Nosal et al., 1991; McNeil et al., 2015). Therefore, an understanding of the molecular mechanisms underlying lysosomal activity and mast cell degranulation is important for the discovery of beneficial therapeutic targets and innovative drug development for the treatment of anaphylaxis.

Our results suggest that dTCTP is an important molecular biomarker that promotes the antigen-induced anaphylactic reactions and that dTBP2 may be effective in treating anaphylaxis through inhibition of the dTCTP-mediated autocrine activation pathway in mast cells.

## Data Availability

The original contributions presented in the study are included in the article/supplementary files, further inquiries can be directed to the corresponding authors.
